# Partial central diabetes insipidus during lithium use: A case report and literature review

**DOI:** 10.1002/pcn5.70182

**Published:** 2025-08-13

**Authors:** Mizue Ichinose, Yuri Kobayashi, Yuhei Suzuki, Yoichiro Hirata, Masayuki Goto, Sho Horikoshi, Keiko Kanno‐Nozaki, Kenya Watanabe, Satoshi Takeuchi, Itaru Miura

**Affiliations:** ^1^ Department of Neuropsychiatry Fukushima Medical University School of Medicine Fukushima Japan; ^2^ Department of Neuropsychiatry Hoshigaoka Hospital Koriyama Japan; ^3^ Department of Psychiatry Itakura Hospital Fukushima Japan; ^4^ Department of Neuropsychiatry Shimizu Hospital Fukushima Japan; ^5^ Department of Psychiatry Horikoshi Psychosomatic Clinic Fukushima Japan; ^6^ Department of Pharmacy Fukushima Medical University Hospital Fukushima Japan

**Keywords:** arginine vasopressin, central diabetes insipidus, lithium, nephrogenic diabetes insipidus, polyuria

## Abstract

**Background:**

Nephrogenic diabetes insipidus (NDI) is a well‐known adverse effect of lithium, which occurs in approximately 20%–40% of long‐term lithium users. Although rare, there have been reports of central diabetes insipidus (CDI) associated with lithium use. Herein, we report a patient with suspected CDI associated with chronic lithium therapy. Furthermore, we conducted a literature search for cases with CDI and discuss the pathogenesis of this case based on previous reports.

**Case Presentation:**

The patient was a 73‐year‐old man with bipolar disorder Type I. His psychiatric symptoms had been stable for many years. However, polyuria and weakness began to appear at the age of 73. Initially, lithium‐induced NDI was suspected, but in the end, partial CDI was suspected because urinary osmolality did not exceed 300 mOsm/L even after water restriction, and administration of nasal arginine vasopressin solution partially increased urinary osmolality.

**Conclusion:**

We have experienced a case in which CDI may have been induced by lithium. Although the effects of ageing and infection cannot be ruled out, it should be noted that when lithium‐induced diabetes insipidus is suspected, CDI may also occur depending on the clinical context.

## BACKGROUND

Lithium is the gold standard drug for the treatment of bipolar disorder. However, its effective blood concentration range is narrow, and caution should be exercised at high doses due to the increased risk of side effects.^1^, ^2^ Nephrogenic diabetes insipidus (NDI) is known to occur in approximately 20%–40% of long‐term lithium users.^3^ It is a well‐known adverse effect of lithium, and many prior reports have addressed this issue. Although rare, there have been reports of central diabetes insipidus (CDI) associated with lithium use.^3^, ^4^, ^5^, ^6^, ^7^ Prior reports suggest that lithium may selectively inhibit cyclic adenosine monophosphate (cAMP) formation in the posterior pituitary gland, preventing antidiuretic hormone vesicle exocytosis, which may lead to CDI.^3^ However, the number of reports is limited, and the mechanism linking lithium to CDI remains unclear.

Herein, we report a patient with suspected partial CDI associated with chronic lithium therapy. Furthermore, we discuss the pathogenesis of this case based on previous reports.

## CASE PRESENTATION

The patient was a 73‐year‐old man with bipolar disorder Type I and a 56‐year history of illness. His medical history included dyslipidemia, and there was no relevant family history. He had been taking lithium (Li) 1000 mg/day and levomepromazine 50 mg/day for more than 30 years. His psychiatric symptoms had been stable for many years; however, polyuria and weakness began to appear in October at the age of 73. A blood sample taken at our hospital showed worsening renal function, with creatinine (Cre) 1.18 mg/dL and Li 1.0 mmol/L, raising suspicion of nephrotoxicity due to lithium. Consequently, the lithium dose was tapered from 1000 to 400 mg/day; however, a month later, he fell at home, became immobile, and was transported to our hospital.

The initial vital signs were as follows: initial blood pressure, 109/92 mmHg; heart rate, 68 beats per minute; oxygen saturation, 97%; and body temperature, 38.7°C. Physical examination revealed swelling and burning in the right lower leg, along with marked thirst. Initial laboratory data indicated dehydration and an elevated inflammatory response; BUN 28.4 mg/dL, Cre 1.20 μg/mL, Li 0.5 mmol/L, WBC 12.1 × 10³/μL, Neutro 92.2%, CRP 42.74 mg/dL, Na 131 mmol/L, K 4.1 mmol/L, Cl 101 mmol/L, BS 118 mg/dL, and HbA1c 5.0%. Radiography, electrocardiography, and head computed tomography (CT) showed no abnormalities, and the right lower extremity symptoms were diagnosed as right lower leg cellulitis. Initial urinalysis revealed no abnormal findings; however, the urine specific gravity was at the lower limit of the normal range. From the subsequent day onward, repeated assessments demonstrated persistently hypotonic urine with a specific gravity of less than 1.005. Daily urine output ranged between 3000 and 6000 mL throughout the hospitalization period.

After admission, the cellulitis gradually improved with antibiotics. The dehydration and polyuria were suspected to be caused by lithium‐induced NDI, so lithium was completely discontinued. Although dehydration was corrected with intravenous fluids after admission, the sodium level rose to 150–152 mmol/L by Day 3 of hospitalization. Additionally, urine output consistently exceeded 4000 mL/day and remained hypotonic. Eight days after admission, the patient developed a duodenal ulcer and was transferred to the department of gastroenterology for treatment. The patient was not taking any medications such as non‐steroidal anti‐inflammatory drugs that could increase the risk of bleeding, and although the cause of the duodenal ulcer was difficult to identify, it was considered possible that stress from inflammation had an impact.

**Figure 1:**
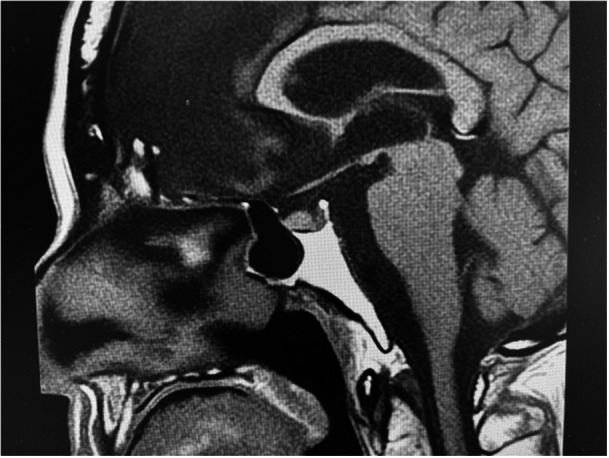
Magnetic resonance imaging (MRI) revealed loss of signal from the posterior pituitary gland.

Polyuria (3000–4000 mL/day) and elevated sodium levels (150–160 mmol/L) persisted after transfer to the department. Magnetic resonance imaging (MRI) revealed loss of signal from the posterior pituitary gland (Figure 1). Based on these findings, the patient was referred to the neurosurgery department for further evaluation on hospital Day 52.

Laboratory findings revealed a urine osmolality of 137 mOsm/kg (50–1300) and urinary sodium of 22 mEq/L (80–250). The plasma arginine vasopressin (AVP) level was 1.4 pg/mL (>1.0 pg/mL), which was low despite continued elevated sodium levels. After intranasal administration of 5 µg of desmopressin, the patient's urine osmolality increased to 229 mOsm/kg. However, the extent of the increase was limited.

He was treated with desmopressin 240 µg/day because of a partial response to AVP nasal spray. Eventually, urine osmolality increased to 230–350 mOsm/L, AVP levels increased to 3.6 pg/mL, and urine volume settled and stabilized at approximately 1500 mL/day.

On the other hand, the patient's mental state became unstable after lithium was discontinued. The patient became violent, and aripiprazole, olanzapine, and valproic acid were administered; however, all were largely ineffective. Ultimately, psychiatric stability was attempted using a combination of haloperidol, loxapine, and carbamazepine, but the patient continued to experience fluctuations in mental state and had difficulty controlling mood symptoms. Subsequently, the patient developed aspiration pneumonia secondary to catatonia. Despite antibiotic treatment, respiratory status worsened, and the patient eventually passed away.

## DISCUSSION

CDI is classified into complete and partial types. In CDI, all three major symptoms—(1) thirst, (2) polydipsia, and (3) polyuria—are present. The diagnostic criteria based on laboratory findings include the following: (1) urine output exceeding 3000 mL per day; (2) urine osmolality below 300 mOsm/kg; and (3) during a water deprivation test, urine osmolality does not exceed 300 mOsm/kg, and after vasopressin administration, urine volume decreases and urine osmolality increases to above 300 mOsm/kg. Furthermore, partial CDI is defined by a relative decrease in AVP secretion in relation to the serum sodium concentration. In complete CDI, the increase in urine osmolality after vasopressin administration is reported to exceed 50%, whereas in partial CDI, the degree of increase is lower.^8^ In the present case, all three major symptoms were observed, and hypotonic polyuria with urine osmolality below 300 mOsm/kg was confirmed. Additionally, urine osmolality after nasal administration of desmopressin showed only a partial increase. Other findings, such as loss of signal in the posterior pituitary on T1‐weighted MRI, low plasma AVP concentration despite hypernatremia, and failure of urine osmolality to exceed 300 mOsm/L after water restriction, led to the diagnosis of partial CDI.

Common causes of CDI include family history, brain tumors, trauma, and infection, but it can also be idiopathic.^9^ In this case, we ruled out a family history, brain tumors, and trauma. On admission, the patient had severe inflammation due to cellulitis; however, polyuria and thirst appeared earlier. Certainly, the possibility of idiopathic onset related to senescence cannot be excluded; however, chronic lithium administration was the most plausible etiology for the development of CDI in this patient. Furthermore, given that laboratory data on admission revealed polyuria accompanied by hyponatremia, concomitant psychogenic polydipsia before hospitalization cannot be definitively ruled out. However, despite the implementation of fluid restriction post‐admission, persistent hypotonic polyuria and hypernatremia were observed, thereby rendering psychogenic polydipsia an unlikely etiology for the sustained polyuria after hospitalization. The suggested mechanism for the pathogenesis of lithium‐induced NDI is that lithium inhibits cAMP formation in the renal tubules and downregulates aquaporin2 (AQP2) receptors in the principal cells of the collecting duct.^10^ However, reports on the association between lithium and CDI are limited. PubMed, PMC, and ICHUSHI were searched for articles published until March 31, 2024. Cases reported in the literature regarding lithium‐associated CDI are presented in Table 1, including our case. CDI develops due to a defect in AVP synthesis or release in the hypothalamus. AVP release from the posterior pituitary is reportedly dependent on cAMP stimulation.^11^, ^12^

**Table 1:** Cases reported in the literature regarding lithium‐associated central diabetes insipidus (CDI).

Reference number	3	4	6	7	The present case
Number of individuals	1	1	1	1	1
Gender/years	Female/31 years	Female/70 years	Female/55 years	Female/73 years	Male/73
Prior psychiatric diagnoses	Bipolar I	Bipolar	Bipolar	Bipolar	Bipolar I
Lithium dosage/duration	300 mg/day	600 mg/day	Unknown	800 mg/day	1000 mg/day
	3 years	3 years	40 years	1 month	30 years
T1‐weighted MRI	No abnormality	Unknown	Decreased signal from the posterior pituitary	Loss of signal from the posterior pituitary	Loss of signal from the posterior pituitary
Reported disease states	CDI	Transient CDI in the setting of underlying chronic NDI	ADH insufficiency associated with ADH depletion due to HHS and NDI	CDI	CDI

Abbreviations: ADH, antidiuretic hormone; HHS, hyperosmolar hyperglycemic syndrome; MRI, magnetic resonance imaging; NDI, nephrogenic diabetes insipidus.

Since lithium inhibits cAMP formation in the tubules, recent reports suggest that lithium‐related CDI may result from the selective inhibition of cAMP formation in the posterior pituitary gland by lithium.^3^, ^4^ Additionally, other reports suggest that prolonged AVP release from the posterior pituitary gland can lead to AVP depletion, resulting in reduced brightness of the posterior pituitary gland on MRI‐T1 images.^13^, ^14^ In the case reported by Gobaru et al., it was suggested that lithium‐induced renal enuresis, complicated by hyperosmolar hyperglycemic syndrome, caused AVP depletion and secondarily induced central enuresis.^6^ Similarly, in the present case, lithium‐induced renal enuresis may have contributed to decreased renal sensitivity to AVP, leading to chronic secretory stimulation of the posterior pituitary gland. Inflammation and dehydration may have further compounded this pathology, ultimately resulting in CDI. Although age‐related idiopathic diabetes insipidus cannot be ruled out, when lithium‐induced diabetes insipidus is suspected, it should be noted that, although rare, CDI may also be induced depending on the clinical context.

## CONCLUSION

We experienced a case in which central enuresis may have been induced by lithium. Based on previous case reports, including this case, it should be noted that if lithium‐induced diabetes insipidus is suspected, although rare, CDI may also be induced in some circumstances.

## AUTHOR CONTRIBUTIONS

Mizue Ichinose conceptualized the idea for this case report. All authors provided review and editing of the manuscript.

## CONFLICT OF INTERESTS STATEMENT

Dr. Miura has received honoraria for lectures from Daiichi Sankyo, Eisai, Janssen, Kowa, Lundbeck, Meiji Seika Pharma, Mochida, MSD, Mylan, Otsuka, Pfizer, Sumitomo Pharma, Takeda, Tanabe Mitsubishi, Towa, Viatris, and Yoshitomi. Dr. Horikoshi has received honoraria for lectures from Eli Lilly, Sumitomo Pharma, Janssen, Meiji Seika Pharma, Viatris, Otsuka, Takeda, and Lundbeck. Dr. Ichinose has received speaker's honoraria from Janssen, Sumitomo, and Otsuka. Dr. Suzuki has received speaker's honoraria from Janssen, Sumitomo, and Otsuka. Drs Kobayashi, Hirata, Goto, Takeuchi, Kanno‐Nozaki, and Watanabe declare no conflict of interest.

## ETHICS APPROVAL STATEMENT

This is a case report that does not require ethics committee approval.

## PATIENT CONSENT STATEMENT

A written informed consent to submit this case report for review and publication was obtained from the patient.

## CLINICAL TRIAL REGISTRATION

N/A.

## Data Availability

The data that support the findings of this study are available on request from the corresponding author. The data are not publicly available due to privacy or ethical restrictions.
